# Ascidian gene regulation and bioadhesion

**DOI:** 10.1002/dvg.23572

**Published:** 2023-11-27

**Authors:** Ute Rothbächer

**Affiliations:** ^1^ Research unit Evolutionary Developmental Biology Institute of Zoology, University Innsbruck Austria; ^2^ Detachement CNRS Marseille France

**Keywords:** adhesive glue, bioadhesion, cis‐regulatory mechanisms, collocytes, evolutionary conservation, gene regulation, sensory adhesive organ

During my university studies in Munich, Germany, I explored Zoology, Biochemistry, Parasitology, and Immunology to focus on tumor biology and melanoma formation in my Diploma and PhD projects in Judy Johnson's lab. She encouraged, guided, and provided maximal freedom for scientific thinking and all basic methods.

Cell specification and the plasticity of cell fate in response to surrounding signals and the resulting precise gene activation/repression mechanisms remain my strong interest. At the end of my PhD I came to three major conclusions: first, we cannot fully understand a pathological situation without knowing in depth about the normal genesis of cells along development; second, we need to study molecular mechanisms *in vivo* to avoid cell lineage artifacts; and third, we need to simplify things by using less complex but informative model organisms that can reveal evolutionarily conserved concepts.

For my post‐doc, I chose *Xenopus* as an *in vivo* model at UC Irvine (Prof. Ken Cho lab) and Caltech Pasadena (Prof. Scott Fraser lab) to reveal conserved molecular players in embryonic signaling, notably that both *Drosophila* and *Xenopus* Dishevelled (Dsh) can mediate Wnt signaling in *Xenopus* secondary axis (Spemann's Organizer) formation (Rothbächer et al., 1995; Rothbächer et al., 2000). We also showed that non‐canonical planar cell polarity signaling via Dsh controls gastrulation in vertebrates (Wallingford et al., 2000) while the canonical ß‐catenin from *Hydra* could induce complete secondary axes upon mRNA injection in *Xenopus* embryos (Hobmayer et al., 2000).

During my postdoc time, my daughter was born and taught me the true miracles of life, also straightening out my priorities and my efficiency. Together, we thereafter moved to Marseille, France.

At that time tunicates (ascidians) were being established in Patrick Lemaire's lab at the Marseille Institute of Developmental Biology as a simpler chordate developmental model, and I soon realized that ascidians could give access to many questions that were rather difficult to address in *Xenopus*. As invertebrate chordates, their larvae resemble an evolutionary prototype for vertebrates! Transparency, few and large cells, and an invariant developmental lineage seemed truly amazing, in addition to techniques like electroporation *en masse* to allow for functional genomics in synchronized embryos. Here, I learned and co‐developed many tools for *Ciona* functional genomics and I worked in collaboration with this lab for around 10 years while publishing my independent research work. Here, I also obtained the “habilitation to direct research” and supervised doctoral candidates. Discovering the earliest zygotic events and the regulatory DNA (enhancer) level of maternally activated target genes was my main interest in ascidians, and we revealed for example, that a GATA factor is required to specify ectodermal cells, and its range of action is restricted by ß‐catenin (Rothbächer et al., 2007).

**FIGURE 1:**
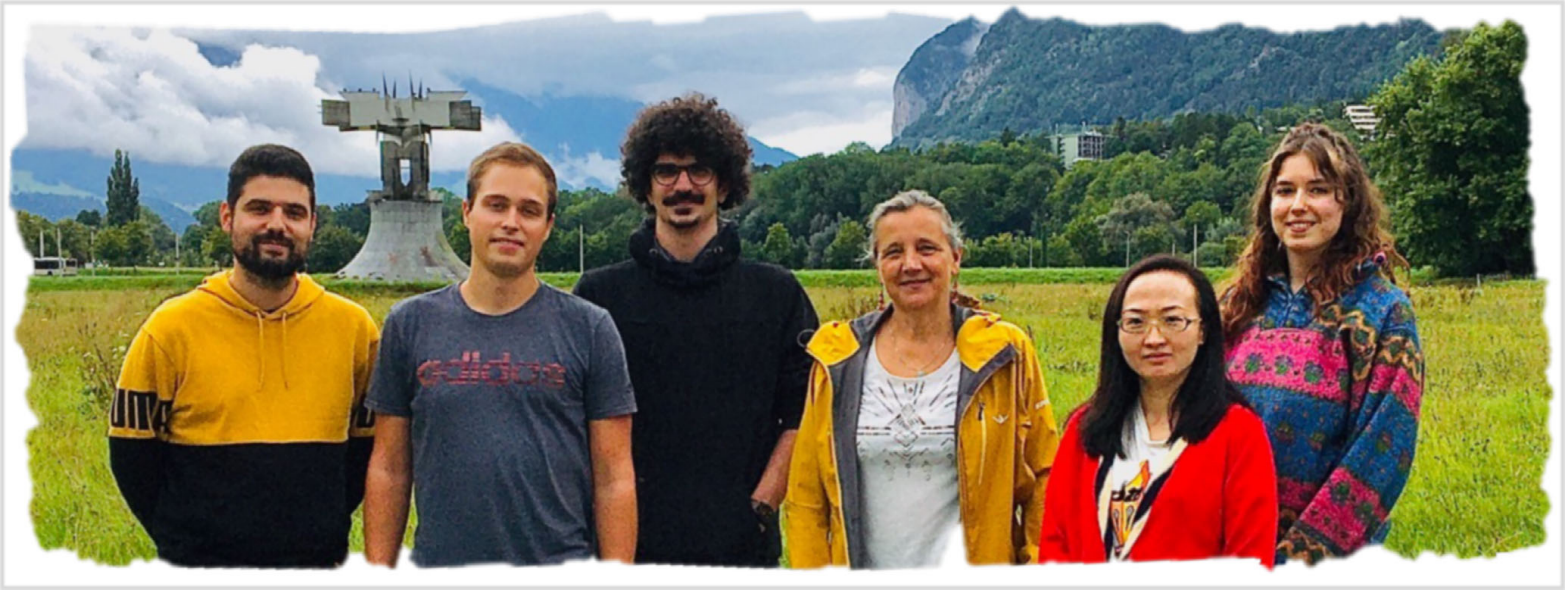
The Innsbruck Tunicate lab today: from left to right, Alessandro Pennati (PhD candidate), David Feldmann and Luca Ciampa (Master students), Ute Rothbächer (group leader), Fan Zeng (postdoc) and Laura Kaczmarek (rotation student).

**FIGURE 2:**
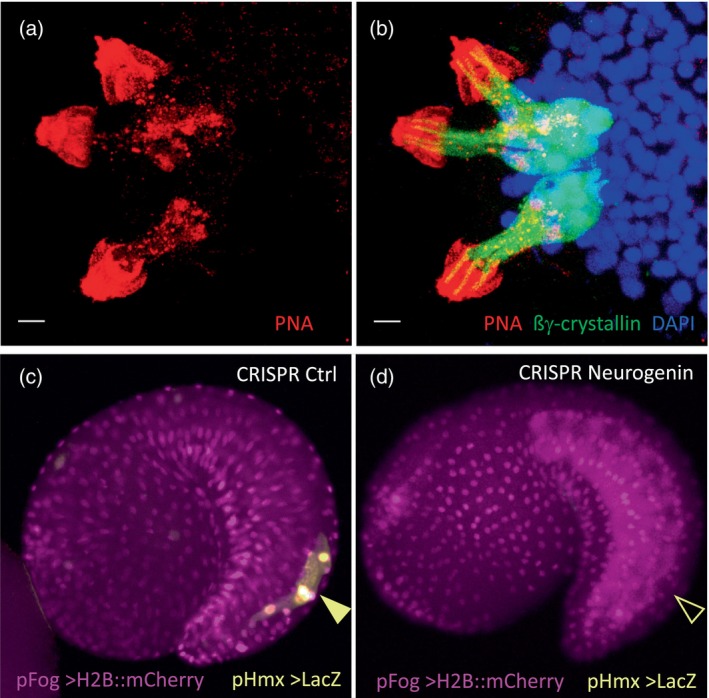
(a,b) Three sensory adhesive papillae of a *Ciona* larva show collocytes and the anterior hyaline cap (to the left) containing adhesive material marked by peanut agglutinin (PNA, red), while (b) axial columnar cells (ACCs) are marked by an anti‐ßγ‐crystallin (green) antibody (Zeng, Wunderer, Salvenmoser, Hess, et al., 2019). Scale bars 5 μm. (c) A control CRISPR electroporated *Ciona* tadpole shows normal expression of the *Hmx* cis‐regulatory region driving expression of the reporter gene lacZ in bipolar tail neurons (BTNs), (d) CRISPR knockout of Neurogenin abolishes expression of Hmx that is required for bipolar tail neuron specification, a sensory cell population that shares Hmx gene expression with vertebrate cranial sensory ganglia of placodal origin. The conservation of cis‐regulatory elements suggests a shared evolutionary ancestry of these cell types (Papadogiannis et al., 2022).

In 2012, I established an independent tunicate research group at the University of Innsbruck, Austria. I have a high teaching load but enjoy mentoring young researchers, and also encourage them to develop new experimental approaches in *Ciona* (Kari et al., 2016). We continued studying the repressive mechanism of ß‐catenin on GATA (Oda‐Ishii et al., 2016), which is reminiscent of opposite wnt/ß‐catenin signaling in *C. elegans* (Murgan et al., 2015). In parallel, inspired by neighboring groups and the technical possibilities offered by *Ciona*, I designed a new project aimed at using our knowledge of bioadhesion in ascidians to develop biomimetic glues (Davey et al., 2021). Primarly conducted by a female reseacher, Fan Zeng, first as a Ph.D. student and currently as a postdoc in my lab (Figure 1), we described in detail the cells within the *Ciona* sensory adhesive papillae (Figure 2a,b; Zeng, Wunderer, Salvenmoser, Ederth, et al., 2019) as well as adhesive components and markers of the adhesive material that these cells secrete at the time of larval settlement (Zeng, Wunderer, Salvenmoser, Hess, et al., 2019), and collaborated with another group on the specification of papillar cells (Johnson et al., 2023). Presently, we are determining the molecular composition of the larval glue in three ascidians and performed *Phallusia* long‐read genome sequencing to resolve repeated regions (unpublished). Through novel CRISPR technology, Ph.D. candidate Alessandro Pennati (Figure 1) identified and characterized conserved cis‐regulation of tail sensory neuron genes (Figure 2c,d) (Papadogiannis et al., 2022). He is now working on an additional repressive mechanism that further refines binary cell fate choice. Sadly, our work on ascidians is increasingly affected by the warming seas, notably by a seemingly shortened reproductive season in both the Northern Altlantic and the Mediterranean Sea.
